# Household

**Published:** 1890-04

**Authors:** 


### HOUSEHOLD.

Gargle for Sore Throat.—A good gargle for sore throat is the following:—Vinegar, one wineglassful; honey, two tablespoonfuls; water, half a tumblerful. Pour the water hot on to the honey, and stir it up; add the vinegar and use cold.

Corn Plaster.—Take of purified ammonia and yellow wax, each 2 oz.; acetate of copper, 6 drachms; melt the two first together over the fire, and after removing from the fire, add the verdigris just before it grows cold. Spread the mixture on soft leather or linen; pare away the corn and apply the plaster. Keep it on a fortnight and then renew it.

Why Quacks Prosper.—A prosperous quack was asked by a doctor how it was he had so many customers. The quack took him to his window overlooking a crowded street, and said, "What proportion of the people passing do you think are sensible persons, with well balanced minds?" "Perhaps one in ten," was the rejoinder. "Just so," said the quack, "and *I get the nine.*"

Orange Syrup.—Orange syrup is so easily made and so convenient to have on hand for various uses, that it is strange more housekeepers do not make it, especially in the season when oranges are plentiful and cheap. Ripe and thin skinned fruit is the best for the purpose. Squeeze the juice through a sieve and to every pint add a pound and a half of powdered sugar with a little of the grated orange peel and the juice of one lemon. Boil the syrup for fifteen minutes, and skim as long as any scum rises. If it does not look clear when taken off, strain it. Next bottle and seal up tight, and it will keep for a long time. Two tablespoonfuls of the syrup mixed with a quarter of a pound of creamed butter makes a nice sauce for a pudding, or a pleasant flavor for custards and ices. Mixed with cold water and ice it makes a delicious drink and can be safely given to invalids.

To Restore the Freshness of Worn Clothing.—Take, for instance, a shiny old coat, vest, or pair of pants of broadcloth, cassimere, or diagonal. The scourer makes a strong, warm soap suds, and plunges the garment into it, souses it up and down, rubs the dirty places, if necessary puts it through the second suds, then rinses it through several waters, and hangs it to dry on the line. When nearly dry, he takes it in, rolls it up for an hour or two, and then pressed it. An old cotton cloth is laid on the outside of the coat, and the iron passed over that until the wrinkles are out; but the iron is removed before the steam ceases to rise from the goods, else they would be shiny. Wrinkles that are obstinate are removed by laying a wet cloth over them, and passing the iron over that. If any shiny places are seen, they are treated as the wrinkles are; the iron is lifted, while the full cloud of steam rises, and brings the nap up with it. Cloth should always have a suds made especially for it, as if that which has been used for white cotton or woolen clothes, lint will be left in the water, and cling to the cloth.

